# Gated recurrent unit-based heart sound analysis for heart failure screening

**DOI:** 10.1186/s12938-020-0747-x

**Published:** 2020-01-13

**Authors:** Shan Gao, Yineng Zheng, Xingming Guo

**Affiliations:** 1 Key Laboratory of Biorheology Science and Technology, Ministry of Education, College of Bioengineering, Chongqing University, Chongqing, 400044 China; 2 Department of Radiology, The First Affiliated Hospital of Chongqing Medical University, Chongqing, 400016 China

**Keywords:** Heart sound, Heart failure screening, Deep learning, Gated recurrent unit

## Abstract

**Background:**

Heart failure (HF) is a type of cardiovascular disease caused by abnormal cardiac structure and function. Early screening of HF has important implication for treatment in a timely manner. Heart sound (HS) conveys relevant information related to HF; this study is therefore based on the analysis of HS signals. The objective is to develop an efficient tool to identify subjects of normal, HF with preserved ejection fraction and HF with reduced ejection fraction automatically.

**Methods:**

We proposed a novel HF screening framework based on gated recurrent unit (GRU) model in this study. The logistic regression-based hidden semi-Markov model was adopted to segment HS frames. Normalized frames were taken as the input of the proposed model which can automatically learn the deep features and complete the HF screening without de-nosing and hand-crafted feature extraction.

**Results:**

To evaluate the performance of proposed model, three methods are used for comparison. The results show that the GRU model gives a satisfactory performance with average accuracy of 98.82%, which is better than other comparison models.

**Conclusion:**

The proposed GRU model can learn features from HS directly, which means it can be independent of expert knowledge. In addition, the good performance demonstrates the effectiveness of HS analysis for HF early screening.

## Background

Heart failure (HF) has attracted widespread attentions due to the high morbidity and mortality, especially with the aging of population. The risk indicators of HF are numerous and complicated. Beside the well-known factors, like obesity, smoking and alcohol abuse, some cardiovascular diseases such as hypertension, earlier heart attack and myocardial infarction have also been verified as the precursors for HF developing in clinical practice [1, 2]. Therefore, keeping a healthy lifestyle and paying attention to the early screening of HF play an important role in the preventive and timely treatment.

HF can be divided into two categories—HF with reduced ejection fraction (HFrEF) and HF preserved ejection fraction (HFpEF), and the following conditions are often used to diagnose of HFrEF and HFpEF in clinical [3]: (1) typical symptoms and/or signs of HF; (2) the indicator of left ventricular ejection fraction; (3) the levels of natriuretic peptides; (4) relevant structural heart disease or diastolic dysfunction. However, these common ways have their own limitations. For instance, the symptoms or signs may be non-specific in the early stages of HF [3], and the invasive measurement [4, 5] is not suitable for promotion among people. The insufficiency in the existing methods prompted us to explore new measures for HF screening.

Nowadays, the non-invasive methods are widely explored for the detection of cardiovascular diseases. For instance, Gao et al. [6, 7] utilized the elasticity-based and a nonlinear state-space approaches to track the motion of carotid artery wall which can be used in the status evaluation of atherosclerotic disease. Many studies used the electrocardiograph signals for cardiac arrhythmia detection [8, 9]; however, the cardiac contractility may not be reflected by electrocardiograph, whose variation is an important sign of HF [10]. Heart sound (HS) can reflect the mechanical dysfunctions of myocardial activity directly, which is a non-stationary physiological signal produced by the beat of muscles [11]. In addition, HS analysis is another non-invasive method. Zheng et al. [12] built a HS-based computer-assisted model in distinguishing HF patients and normal by analyzing the cardiac reserve.

In traditional HS analysis, the feature extraction and/or selection is a crucial step, and various features have been used in HS field, such as wavelet transform [13], wavelet packet transform [14], energy entropy [15] and Mel-frequency cepstral coefficients [16]. These features may be more intuitive to reflect the physical meaning of HS in different states. However, three main limitations also exist: (1) feature extraction and/or selection depends largely on professional knowledge in the fields of medicine and signal processing; (2) extraction of hand-crafted features may miss valuable deep features which contain the latent information of HS; (3) some hand-crafted features are ineffective when the sample quality varies greatly [17]. Deep learning methods, as the new field in machine learning, can learn the features automatically from the inputs without the process of hand-crafted feature extraction and have become popular in the field of biomedical. A convolutional neural network-based transfer learning approach is proposed by Zhang et al. [18] for automatic colorectal cancer diagnosis. Gao et al. [19] proposed a novel deep neural network to learn the implicit strain reconstruction from 2D-radio frequency images and assess the conditions of disease. However, these models have limited ability to mine the features from time-series signals. The improved recurrent neural networks (RNN), including long short-term memory (LSTM) and gated recurrent unit (GRU), can keep the relation of input sequences; therefore, they have been successfully used in sequential data prediction or classification. Yu et al. [20] have adopted the LSTM with attention mechanisms to predict the patient mortality in hospital. Vetek et al. [21] applied LSTM to classify temporal sleep stage using several physiological signals. Similar studies based on EEG were tested by Michielli [22]. Xu et al. [23] reported a LSTM-based architecture for motion-feature extraction from the region of interest sequences. Although RNN-based networks have been extensive used and gained resounding success in biomedical sequence processing, they are barely applied in HS classification.

**Fig. 1:**
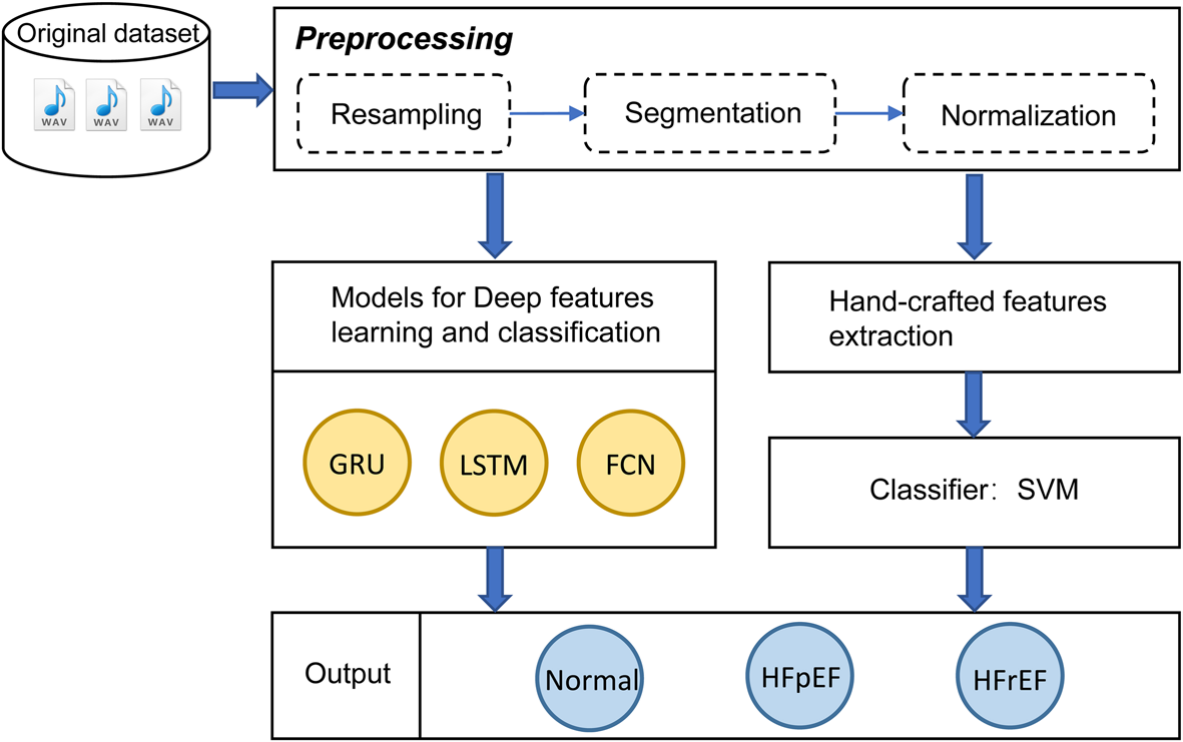
The illustration of the workflow of this paper. The GRU is the proposed model while others are the methods compared

To address the above issues, we proposed a novel GRU-based method for HF screening using HS. The contributions of this paper lie in: (1) to our best knowledge, this is the first study to distinguish the normal, HFpEF and HFrEF subjects using HS; (2) without heavy reliance on expert knowledge and any hand-crafted features, the proposed method screens HF utilizing HS signals; (3) the performances show that our method is substantially better than two other deep learning models and one traditional features extraction method. The main framework of this paper is depicted in Fig. 1.

## Results

The algorithms of signal preprocessing (resampling, segmentation and normalization), hand-crafted feature extraction and classification with support vector machine (SVM) were all implemented on Matlab (version R2016b) programming. The deep learning models in this work were implemented using python (version 3.5.4) on Tensorflow library (version 1.12.0). The computer used with a 3.7-GHz Intel Core i7-8700 K CPU, GTX 2080Ti GPU with 11 GB video memory and 64 GB RAM to train the networks.

### Model setting experiments

**Fig. 2:**
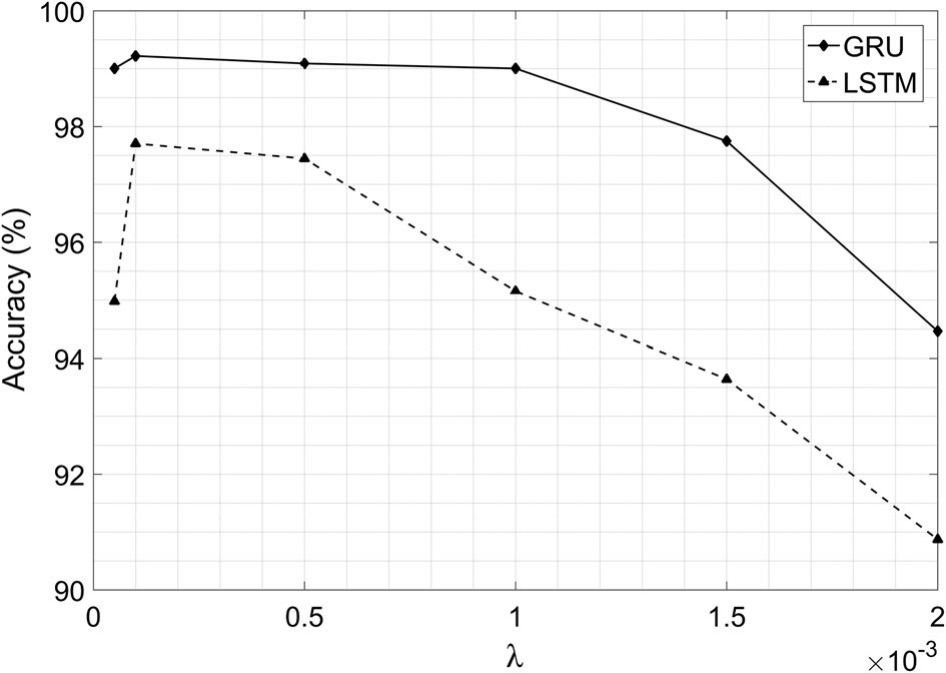
The test accuracy influenced by the weight $\lambda$ of L2 loss. When $\lambda$ is set as 0.0001, the GRU and LSTM both reach the highest accuracy

The basic settings of GRU model are determined as follows: Adam is selected as the optimizer and the learning rate is set as 0.001. Softmax cross entropy with logits v2 is chosen as the main loss function. Besides, L2 norm is added in the loss function to prevent model overfitting [24]. The L2 norm of the weight $\lambda$ for weight decay is calculated by some experiments carefully, and finally set as 0.0001 according to Fig. 2. All the parameters in this paper are trained with the batch size of 64, and the models are trained for 50 epochs in total.

**Fig. 3:**
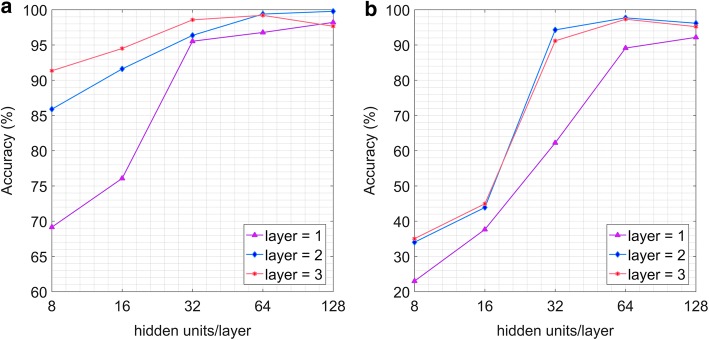
The accuracy comparison between the number of layers and the number of hidden units/layer: **a** GRU; **b** LSTM

**Fig. 4:**
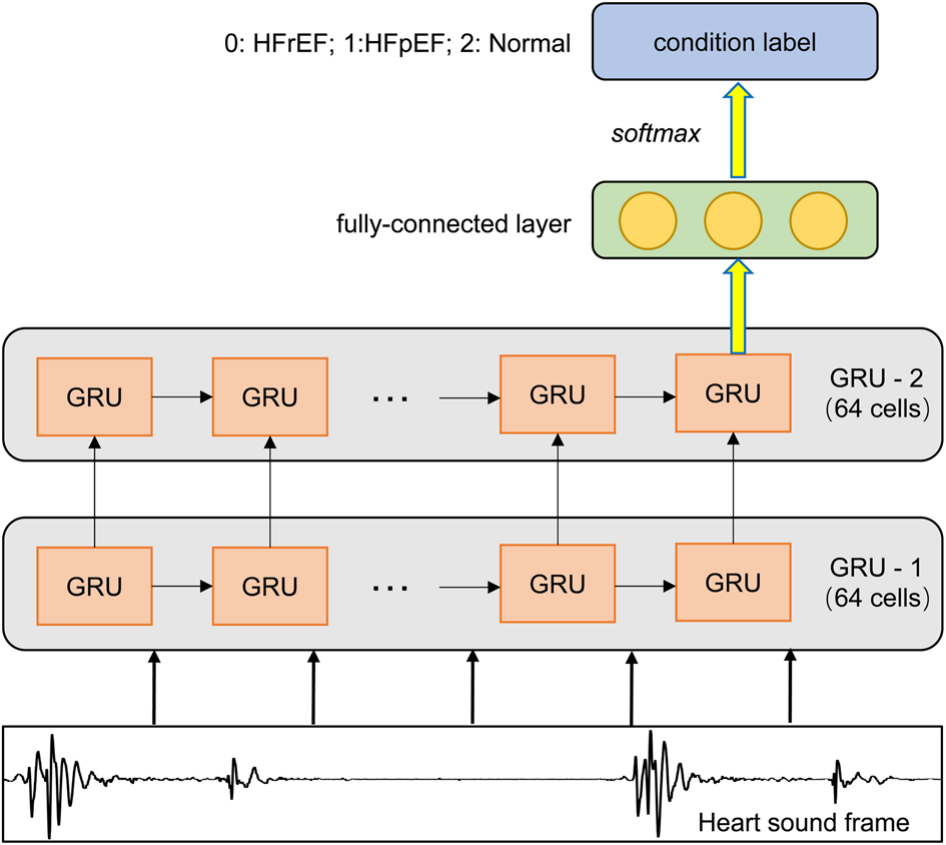
The proposed GRU framework for HF screening. The input of the model is the frame of normalized HS with the length of 960 sampling points. The architecture has two GRU layers with 64 units/layer and a fully connected layer of 3 units (the number of HS categories). The LSTM has the similar framework, but the GRU units are changed to LSTM units

Considering the experimental results about the number of layers and hidden units/layer, the structures of GRU are finally determined. The number of layers varies in {1,2,3}, and the number of units for per layer ranges in {8,16,32,64,128}. As the experimental results show in Fig. 3a, the overall effect of two layers is better than one layer. When the number of units exceeds 64, the performance of three layers is even worse than that of two layers. Considering the complexity of model and the recognition accuracy comprehensively, the GRU structure finally is chosen as two layers with 64 hidden units/layer. Figure 4 shows the final architecture of the GRU network. Moreover, the structure of LSTM is defined the same with that of GRU. Figure 3b exemplifies the relevant experimental results of LSTM.

### Screening performance

To evaluate the robustness and to ensure the repeatability of proposed models, the tenfold cross-validation was used in this work. For each fold, 90% of the HS frames are used for training and the remaining 10% is used to test the performance of our models. To monitor and tune the parameters of training process, 20% frames of the training set are sampled to be used as validation set.

**Fig. 5:**
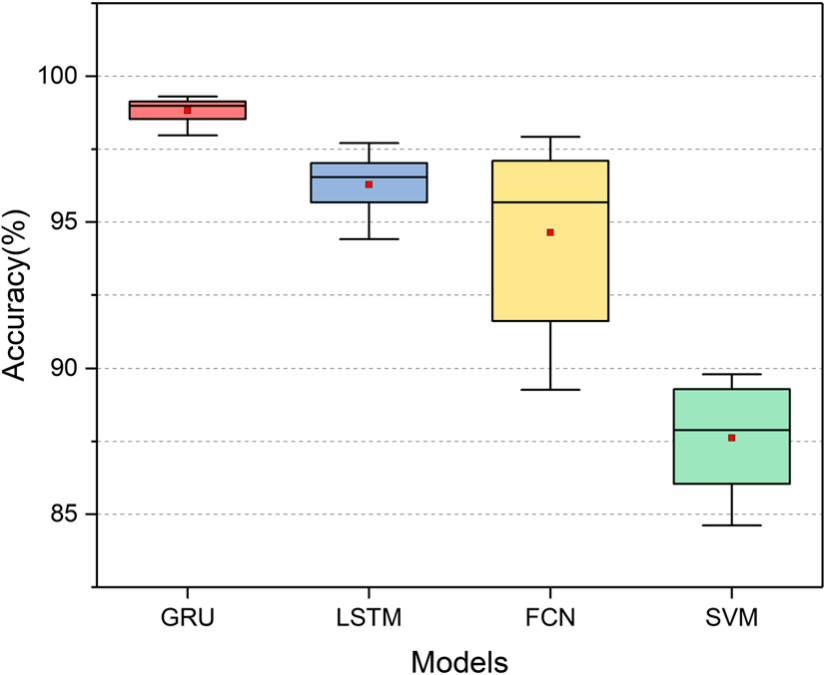
The accuracies of different models with box-plot. The mean value ± standard deviation for these models are:${\text{Acc}}_{\text{GRU}} = 98.82\% \pm 0.46\%$, ${\text{Acc}}_{\text{SVM}} = 87.62\% \pm 1.77\%$, ${\text{Acc}}_{\text{FCN}} = 94.65\% \pm 3.07\%$, ${\text{Acc}}_{\text{LSTM}} = 96.29\% \pm 1.02\%$. Deep features based on GRU model show the highest accuracy on average

The performance of tenfold cross-validation for all methods is summarized in Table 1. It can be seen that GRU achieves the best average accuracy of 98.82%, which is 2.53%, 4.17% and 11.2% higher than LSTM, fully convolutional network (FCN) and SVM, respectively. SVM is the lowest performing model compared with the other three deep learning models. In addition, the performance of the GRU is more stable as the accuracy deviation is the minimum compared with that of the other three models, which is depicted in the box-plot in Fig. 5.

**Table 1:** The tenfold cross-validation results of different models and their average accuracy

Models	Value of accuracy in each fold (%)										Average
	1	2	3	4	5	6	7	8	9	10	
SVM	87.32	*89.80*	86.05	84.62	89.29	87.27	88.52	89.36	88.46	85.48	87.62
FCN	94.38	*97.92*	91.61	89.27	95.29	97.36	96.06	96.76	97.10	90.70	94.65
LSTM	96.97	97.02	94.42	95.68	97.15	96.54	96.54	95.16	*97.71*	95.68	96.29
GRU	99.22	98.92	98.14	97.97	99.09	98.83	99.05	98.53	*99.31*	99.14	*98.82*

The best result is highlighted in italics

**Fig. 6:**
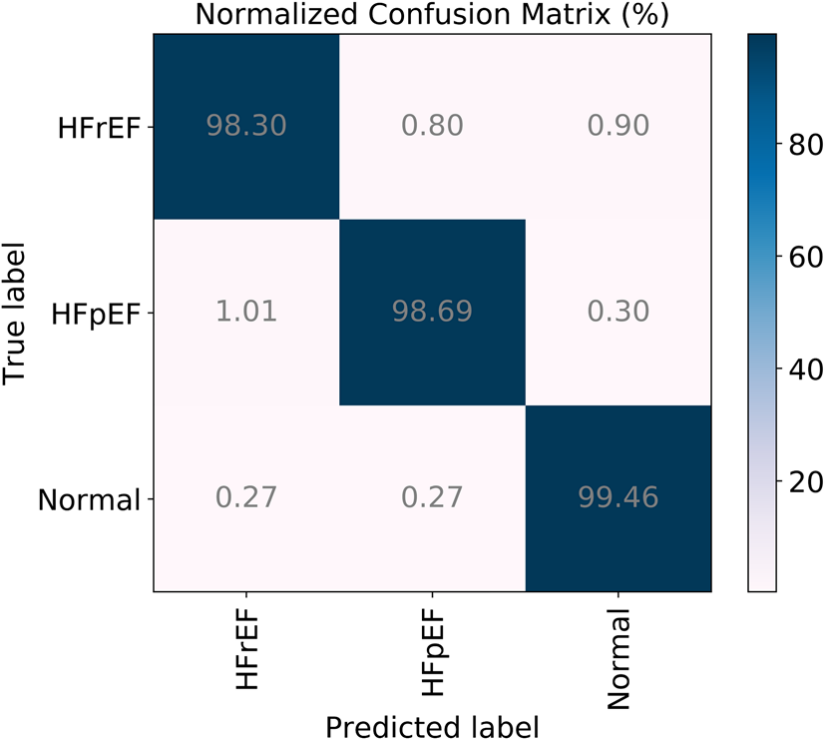
Final normalized confusion matrix of GRU model with all tenfold testing data. The columns of the confusion matrix represent the predicted classes and the rows represent the true classes

Table 2 shows the confusion matrix of GRU with all tenfold testing data. The values of precision in three categories are in the range of 98.7–98.93%, and the values of recall are in the range of 98.31–99.46%. It shows that the proposed GRU model can recognize three classes of HS precisely, in which the accuracy of normal class is recognized best. Figure 6 shows an intuitive normalized confusion matrix.

**Table 2:** A confusion matrix of HF for GRU across all tenfold testing data

	HFrEF	HFpEF	Normal	Recall
HFrEF	7540	61	69	98.30%
HFpEF	78	7609	23	98.69%
Normal	21	21	7698	99.46%
Precision	98.70%	98.93%	98.82%	98.82%

The columns represent the predicted categories and the rows represent the true categories

## Discussion

### The impact of the length of frames on classification results

In this paper, the HS signals were segmented to fixed length (1.6 s) frames, and the length of frames might affect the classification stage. To evaluate the possible effect of frame length on final performance, the experiments with fixed length of 0.8 s (approximately one cycle) frames were explored. The corresponding tenfold cross-validation results using the proposed GRU model are listed in Table 3. The results show that the dataset with 1.6 s frames could obtain the average accuracy about 2% higher than 0.8 s frames. The deviation may be caused by the missing of interval features in one cycle frame, which contributes a lot on the classification stage.

**Table 3:** Tenfold cross-validation results of GRU model with two types of frame length

Frame length	Value of accuracy in each fold (%)										Average
	1	2	3	4	5	6	7	8	9	10	
0.8 s	96.37	97.71	95.37	95.85	97.63	96.19	95.42	96.93	*97.92*	96.71	96.61
1.6 s	99.22	98.92	98.14	97.97	99.09	98.83	99.05	98.53	*99.31*	99.14	*98.82*

The best result is highlighted in italics

### The comparison of the methods used in this study

In this paper, four models were used to compare the performance for HF screening. GRU and LSTM models are modified kind of RNN architectures. Generally, RNN models can achieve better results than others used in this study. It is because the RNN models can keep the relation of the input time series while others cannot [24]. The results of tenfold cross-validation show that GRU model can achieve higher performance than LSTM model in every attempt of HF screening. Moreover, our comparative experiments have proven that deep learning models outperform the SVM in HF screening. As a representative of traditional knowledge-driven methods, the unsatisfactory results of SVM may be related to the selection of features. Additionally, taking HS signals directly as the input, deep learning models can realize automatic classification without any hand-crafted feature extraction or selection; therefore, our model with fine-tuned parameters can also be applied into other signal processing areas. In sum, the deep learning models can get the higher precision and better performance than traditional SVM, especially the proposed GRU model.

### The comparison of the relevant studies

Over the years, many studies on screening of HFrEF and HFpEF have been conducted. However, most of the studies were based upon biochemical indicators, phenotype and statistical analysis of medical records information. For instance, Savarese et al. [25] used N-terminal pro-B-type natriuretic peptide to distinguish different HF category. These biochemical indicators are useful to diagnose HF and predict prognosis in HF, but they play a very limited role in the early screening of HF. In addition, such invasive diagnostic methods are not suitable for pervasive application. Xanthopoulos et al. [26] proposed a method to classify the HFpEF based on the phenotype of hypertension, which requires researchers to have a wealth of medical knowledge.

HS signals are closely related to cardiovascular diseases and have been widely studied, while objects of these researches were different. For example, the identification and classification of HS components [27, 28], classification of normal and other abnormal HS [29–31], differentiating the murmurs between physiological and pathological [32, 33]. However, the previously published papers about classification of HFrEF, HFpEF and normal were few and incomplete. Liu et al. [34] explored the difference between HFpEF and normal, but they omitted the study about HFrEF. Zheng et al. [35] reported a HF identification method using HS; however, the HFrEF and HFpEF were not explored separately. It can be seen that the study on HF screening, which included normal, HFpEF and HFrEF, has not been studied sufficiently. Hence, this study could be an efficient complement for HF screening.

### The limitations and future work of this study

This study has three limitations. Firstly, for the lack of HS databases about HFrEF and HFpEF, the experimental tests for generalization ability on other public databases using our method could not be made. Secondly, experimental method was used for the hyper-parameters setting of GRU and LSTM in this study. This method needs to run many experiments to involve approximating optimal value. In the future work, other methods of tuning parameters like grid search may be used in our model to improve the efficiency. In addition, the normal HS may be quite different from that of HF patients, in order to better verify the performance of the proposed method, the abnormal HS with normal systolic and diastolic function can be considered as the control group in the feature.

## Conclusions

Early screening of HF can provide a timely guide for treatment. In this paper, GRU-based HS analysis method was proposed to screen HF automatically. Taking HS signals as input, the method eliminates the dependence on hand-crafted feature extraction. To verify the screening accuracy, LSTM, FCN and SVM models were carried out as the comparative experiments. The results show that the performance of GRU model is competitive with the methods compared, especially the traditional method of SVM, and it is promising as an effective method for the non-invasive HF screening. In future, the applicability of the method mentioned in this paper will be validated in other cardiovascular diseases, like cardiac murmurs, valvular disease.

## Methods

### Experimental data description

The HS data used in this paper contain three categories—HFrEF, HFpEF and normal. The HS signals of HF patients were acquired from University-Town Hospital of Chongqing Medical University using the HS acquisition system (Patent No.: CN2013093000306700) with the sampling frequency at 11,025 Hz. HF samples were collected from 42 HFrEF and 66 HFpEF patients, respectively. Moreover, all the patients of HFrEF and HFpEF were diagnosed and confirmed by the cardiologists. All patients signed informed consent forms before participating this study, and this study has been ratified by Ethical Commission Chongqing University. The normal HS was obtained from the PhysioNet/Computing in Cardiology Challenge 2016. It contains nine databases from different research groups, and all recordings in the dataset were resampled to 2000 Hz. The dataset includes 2435 normal HS recordings collected from 1297 healthy subjects. Details of the dataset can be referenced in [36, 37]. In this paper, 1286 recordings were randomly selected as the normal group.

### Signal preprocessing

HS preprocessing is an essential part to achieve a good identification performance. In this study, the preprocessing includes three steps introduced as follows.

#### Resampling

In general, HS mainly comprises two components: the first HS (S1) and the second HS (S2). S1 is the transient low-frequency acoustic signals, which is mainly between among 10 and 200 Hz, produced by the vibrations of heart chambers, heart valves and blood in systolic. S2 is produced at the end of systole, following the closure of semilunar valves about aortic and pulmonary [27, 38]. S2 has a higher-pitch than S1, with its frequency range between 20 and 250 Hz [39]. Since the original sampling frequency may cause high computational cost, all recordings are down-sampled at 600 Hz in accordance with Nyquist Sampling Theorem.

#### S1 marking and segmentation

In order to standardize the input length for the model, one strategy was used in this paper to obtain HS frames. Two main steps are involved in this process: marking S1 onset and segmentation HS with fixed frame length.

##### Marking S1 onset

Positioning the boundaries of HS components is the critical operation of segmentation. A cardiac period contains four states, namely S1, systole, S2 and diastole. Since S1 is the start of a cardiac cycle, the S1 onset is considered as the boundary of frames.

In this paper, logistic regression-based hidden semi-Markov model (LR-HSMM) is selected to localize the onset of S1. The method of LR-HSMM, developed by Springer et al. [40] and verified by Liu et al. [36], is usually treated as the state-of-the-art method for HS segmentation or marking the onset of cycles, which has great robustness in processing noisy recordings. To preserve more details of HS, the step of signal denoising was skipped in this study. Thanks to the advantages of LR-HSMM, the onset of S1 can be located accurately as shown with the dotted line in Fig. 2.

##### Segmentation HS with fixed frame length

**Fig. 7:**
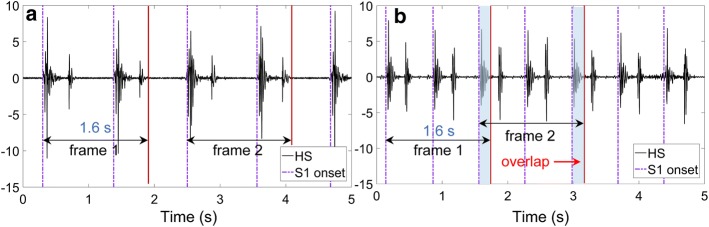
Automatic S1 onset marking using LR-HSMM and period synchronous segmentation into 1.6 s frames. The dotted lines are the S1 onset and the red lines are the end boundaries of frames: **a** is without overlap; **b** is with overlap

The mechanical activity of heart is captured in one cardiac period [41]. Moreover, the interval features may vary between each cycle. In view of these two factors, period synchronous segmentation with the fixed frame length was applied in this study. The duration of a cardiac cycle is about 0.6–0.8 s, thus the frame length is fixed as 1.6 s, which includes approximately two cardiac cycles. Depicted in Fig. 7a, we segmented the frames with an interval of one cardiac cycle. Whenever the frame length exceeds two periods, overlap is inherent, which is exemplified in Fig. 7b. A total of 23,120 HS frames have been segmented, which, respectively, include the frames of HFrEF, HFpEF and normal are 7670, 7710 and 7740.

#### Normalization

Normalization is necessary to eliminate the difference of HS amplitude caused by the differences of acquisition locations and individual variation of subjects [15, 16]. All frames used in this paper were normalized by the following formula:

1 $$X{\kern 1pt} \,{ = }{\kern 1pt} \,\frac{{x - x_{\text{min} } }}{{x_{\text{max} } - x_{\text{min} } }}.$$

### RNN-based structures

RNN models, including LSTM and GRU, were used in this work to learn deep features from HS. In this part, some detailed information about the RNN, LSTM and GRU are described as follows.

#### RNN

Generally, neural networks assume that inputs and outputs are independent from each other, while many relatedness exist between outputs and previous inputs in reality. Different from other deep learning models, RNN is a network with memory capabilities that can be used to process time sequence data. Hidden layers inputs $h^{(t)}$ include both the previous hidden output $h^{(t - 1)}$ and the current input $x^{(t)}$. It can be expressed as:

2 $$h^{(t)} = f(Ux^{(t)} { + }Wh^{{(t{ - 1})}} { + }b),$$

where $U$, $W$ and $b$ represent the input weight, hidden unit weight and bias, severally. RNN networks can mine information from arbitrarily long sequences theoretically, but they are limited to just a few steps in practice. For engineering application, LSTM and GRU, the improved RNN networks, are used widely.

#### LSTM

As an advanced version of general RNN, LSTM was proposed by Hochreiter and Schmidhuber [42] firstly and improved by Graves [43]. It solved the problem of weight explosion or gradient disappearing due to recursion under long-term time correlation conditions.

**Fig. 8:**
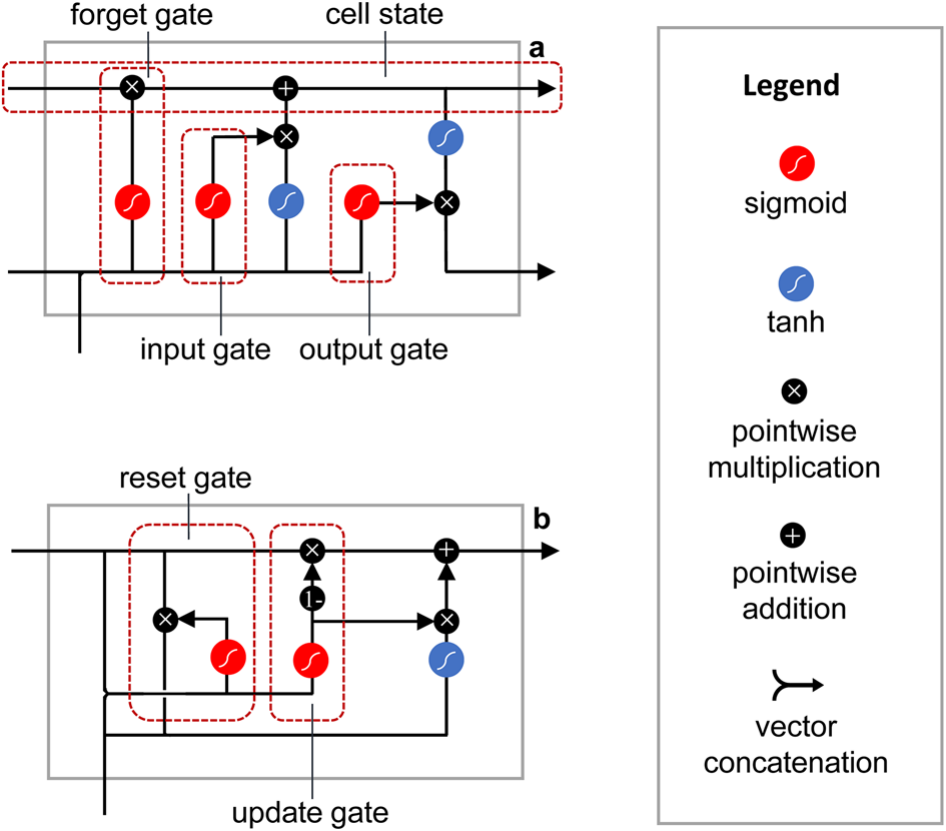
Structures of LSTM unit and GRU unit: **a** is the structure of LSTM unit, including three gates: input gate, forget gate and output gate; **b** is the structure of GRU unit, which is equipped with the reset gate and update gate

The architecture of LSTM contains a cluster of cyclically connected memory cells, and each LSTM unit is equipped with input gate, forget gate and output gate. These gates control the manner of which internal states are retained or discarded. The structure of LSTM unit is shown in Fig. 8a. The algorithm equations of LSTM cell from inputs to outputs are specified as follows:

3 $$g^{(t)} = \sigma (b_{g} + U_{g} x^{(t)} + W_{g} h^{(t - 1)} ),$$

4 $$f^{(t)} = \sigma (b_{f} + U_{f} x^{(t)} + W_{f} h^{(t - 1)} ),$$

5 $$o^{(t)} = \sigma (b_{o} + U_{o} x^{(t)} + W_{o} h^{(t - 1)} ),$$

6 $$s^{(t)} = f^{(t)} s^{(t - 1)} + g^{(t)} \sigma (b + Ux^{(t)} + Wh^{(t - 1)} ),$$

7 $$h^{(t)} = \tanh (s^{(t)} )o^{(t)} ,$$

where the $\sigma$ represents the sigmoid function keeping the weights at 0–1, and $g^{(t)}$, $f^{(t)}$, $o^{(t)}$, $s^{(t)}$ indicate the external input gate, forget gate, output gate and cell state unit, respectively. The $b$, $U$ and $W$ mean the biases, input weights and circular weights, respectively.

Behind the LSTM layers, a fully connected layer with a softmax function is applied for classification. The softmax function is as follows:

8 $${\text{softmax}}(x_{i} ) = \frac{{{ \exp }(x_{i} )}}{{\sum\nolimits_{i} {{ \exp }(x_{i} )} }},$$

where $x_{i}$ is the output of former layer.

#### GRU

GRU, a special variant of the LSTM network, was proposed by Cho et al. [44] in 2014. The structure of the GRU is simplified from the LSTM, with two gates, but not separate memory cell. A single update gate $z^{(t)}$, which replaced the input gate and the forget gate in LSTM, is used to estimate the current state of output. Furthermore, the reset gate $r^{(t)}$ is introduced to control the influence of the previous hidden state on the $x^{(t)}$ directly. The update gate and reset gate are described as below:

9 $$z^{(t)} = \sigma (b_{z} + U_{z} x^{(t)} + W_{z} h^{(t - 1)} ),$$

10 $$r^{(t)} = \sigma (b_{r} + U_{r} x^{(t)} + W_{r} h^{(t - 1)} ),$$

and the state of the hidden layer $h^{(t)}$ is computed as below:

11 $$h^{(t)} = z^{(t)} h^{(t - 1)} + (1 - z^{(t)} )\tilde{h}^{(t)} ,$$

where $\tilde{h}^{(t)} = \tanh (b_{h} + U_{h} x^{(t)} + W_{h} r^{(t)} h^{(t - 1)} )$, $U$, $W$ are the weight matrices of different gate referring to the subscripts, and $b$ represents the bias. Figure 8b gives the structure of GRU unit.

Output states of GRU are calculated using a softmax function (Eq. (8)), which is the same with LSTM.

### Methods compared

*FCN:* FCN with a softmax output layer has been used for time series classification [45]. The model comprised three convolutional blocks with the filter size of 128, 256, 128 and kernel sizes 8, 5, 3, respectively. A batch normalization layer and a ReLU layer are followed by every block. Then the global average pooling layer is added before the softmax layer to reduce the number of weights. The model is trained for 50 epochs with the batch size and learning rate of 64 and 0.001, respectively.

*SVM:* A one-versus-one SVM classifier with radial basis function kernel is adopted. Grid search method is used for parameters tuning. Following Ref. [46], we extracted multiple-type features from HS of HFrEF, HFpEF and normal. Three features with *P*-value less than 0.001 in Tamhane’s T2 one-way ANOVA are chosen as the feature vector for SVM. To ensure the compactness of this paper, the hand-crafted feature selection and analysis are presented in the “Appendix” at the end of the paper.

*LSTM:* A structure with two layers and 64 hidden units/layer is adopted. The details are explained in the results.

*GRU:* Proposed method.

## Figures and Tables

**Fig. 9:**
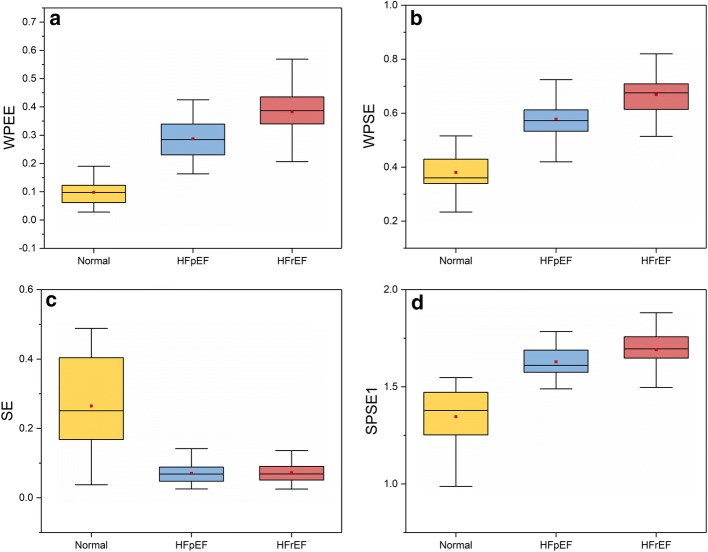
The statistical results for three categories with box-plots. The red dots represent the means, and the midlines in the boxes represents the medians: **a** WPEE, **b** WPSE, **c** SE and **d** SPSE1

## Data Availability

The normal HS database is available on PhysioNet. (https://www.physionet.org/physiobank/database/challenge/2016/). The HFrEF and HFpEF databases are not publicly available due to the interest of National Natural Science Foundation of China.

## Appendix

The hand-crafted features we extracted include wavelet packet energy entropy (WPEE), wavelet packet singular entropy (WPSE), sample entropy (SE) and eight components of sub-band power spectral entropy (SPSE), respectively. For the detailed description of these features, refer to [46]. Tamhane’s T2 one-way ANOVA is adopted for multiple comparisons, which is a reliable pairwise comparison based on independent sample *T*-test. The *P* values of extracted features are presented in Table 4, and the *P*-values of WPEE, WPSE and SPSE1 are less than 0.001, indicating that these three features are significantly different among three categories. The SE has the difference between normal and HF groups, but no difference in HF groups. The rest of the features almost have no differences. Therefore, WPEE, WPSE and SPSE1 are finally chosen as the feature vector for SVM.

**Table 4:** The *P*-values of Tamhane’s T2 one-way ANOVA

Multiple comparison		WPEE	WPSE	SE	SPSE1	SPSE2	SPSE3	SPSE4	SPSE5	SPSE6	SPSE7	SPSE8
HFrEF	HFpEF	< 0.001	< 0.001	0.989	< 0.001	0.191	0.169	0.612	0.067	0.294	0.024	0.066
HFrEF	Normal	< 0.001	< 0.001	< 0.001	< 0.001	0.039	0.387	0.495	0.999	0.006	< 0.001	< 0.001
HFpEF	Normal	< 0.001	< 0.001	< 0.001	< 0.001	< 0.001	0.993	0.992	0.029	< 0.001	0.001	0.001

Figure 9 shows the qualitative results of WPEE, WPSE, SPSE1 and SE using box-plots. The values of WPEE, WPSE, SPSE1 keep the same trends among the three groups, i.e., the normal group is the lowest, while HFrEF group is the highest. These trends indicate the myocardial contractility changes in cardiac energy and information complexity during the development of HF.

## Abbreviations

HF: heart failure
HS: heart sound
GRU: gated recurrent unit
LVEF: left ventricular ejection fraction
HFrEF: heart failure with reduced ejection fraction
HFpEF: heart failure with preserved ejection fraction
SVM: support vector machine
RNN: recurrent neural networks
LSTM: long short-term memory
FCN: fully convolutional network
LR-HSMM: logistic regression-based hidden semi-Markov model
